# The Effect of Fermentation on the Chemical Constituents of Gastrodia Tuber Hallimasch Powder (GTHP) Estimated by UHPLC-Q-Orbitrap HRMS and HPLC

**DOI:** 10.3390/molecules29071663

**Published:** 2024-04-07

**Authors:** Yaning Wu, Hongwei Zhang, Jianguang Zhu, Zhenling Zhang, Songbo Ma, Yongqi Zhao, Yiming Wang, Jun Yuan, Xing Guo, Yajing Li, Shuai Zhang

**Affiliations:** 1 School of Pharmacy, Henan University of Chinese Medicine, Zhengzhou 450046, China; 18839387651@163.com (Y.W.);; 2 Collaborative Innovation Center of Research and Development on the Whole Industry Chain of Yu-Yao, Henan Province, Zhengzhou 450046, China; 3 Henan Engineering Technology Research Center for Integrated Traditional Chinese Medicine Production, Zhengzhou 450046, China; 4 Henan Engineering Research Center of Traditional Chinese Medicine Characteristic Processing Technology, Zhengzhou 450046, China; 5 Luoyang Wokang Pharmaceutical Co., Ltd., Luoyang 471521, China

**Keywords:** UHPLC-Q-Orbitrap HRMS, GTHP, fermentation, chemical composition, fingerprinting

## Abstract

Objective: To compare the effect of fermentation on the chemical constituents of Gastrodia Tuder Halimasch Powder (GTHP), to establish its fingerprinting and multicomponent content determination, and to provide a basis for the processing, handling, and clinical application of this herb. Methods: Ultra-high-performance liquid chromatography-quadrupole-Orbitrap high-resolution mass spectrometry (UHPLC-Q-Orbitrap HRMS) was used to conduct a preliminary analysis of the chemical constituents in GTHP before and after fermentation. High-performance liquid chromatography (HPLC) was used to determine some major differential components of GTHP and establish fingerprints. Cluster analysis (CA), and principal component analysis (PCA) were employed for comprehensive evaluation. Results: Seventy-nine compounds were identified, including flavonoids, organic acids, nucleosides, terpenoids, and others. The CA and PCA results showed that ten samples were divided into three groups. Through standard control and HPLC analysis, 10 compounds were identified from 22 peaks, namely uracil, guanosine, adenosine, 5-hydroxymethylfurfural (5-HMF), daidzin, genistin, glycitein, daidzein, genistein, and ergosterol. After fermentation, GTHP exhibited significantly higher contents of uracil, guanosine, adenosine, 5-hydroxymethylfurfural, and ergosterol and significantly lower genistein and daidzein contents. Conclusions: The UHPLC-Q-Orbitrap HRMS and HPLC methods can effectively identify a variety of chemical components before and after the fermentation of GTHP. This study provides a valuable reference for further research on the rational clinical application and quality control improvement of GTHP.

## 1. Introduction

GTHP is a dried bacterial powder made from *Armillaria mellea* by liquid fermentation and culture, separated and extracted, and it was included in the “Volumes 13 of Chemicals from Local to National Standards” in 2002 [1]. *Armillaria mellea* is rich in terpenoids, polysaccharides, nucleosides, and sterols. Modern pharmacological studies have shown that *Armillaria mellea* can improve insomnia, act as an antidepressant, treat Alzheimer’s disease, and address insufficient blood supply to the vertebral basilar artery [2,3]. The ethyl acetate extract of *Armillaria mellea* showed the activity to inhibit inflammatory mediators [4]. The polysaccharides of *Armillaria mellea* have demonstrated anti-Alzheimer’s disease and hypoglycemic effects [3,5], while the sesquiterpene constituents presented antidepressant effects [3]. In addition, the melleolide components in *Armillaria mellea* have exhibited anti-hepatocarcinogenic and antibacterial effects [6,7]. GTHP is clinically used for neuroprotection, the treatment of tension headaches, and hypoglycemic effects [8].

Traditional fermentation techniques have been used for thousands of years. Products such as white wine, vinegar, and tempeh, which are made through fermentation, continue to be important components of the human diet [9]. Modern research has concluded that fermentation depends on the metabolic activities of microorganisms, with the specific species determining the quality of fermented products [10]. Chinese medicine fermentation technology involves the use of bacterial fermentation metabolism to produce a variety of effective enzymes to change the chemical composition of drugs. This process facilitates the decomposition of certain ingredients into new active components and can also break down toxic elements, thereby reducing the potential toxicity and side effects. In addition, the process can also enhance the therapeutic effect of the resulting products [11]. *Dendrobium officinale* juices were fermented to produce polysaccharides that could be more easily absorbed and enhance the potential function of the polysaccharides. In addition, the fermentation process was conducted to produce gallic acid, a new functional substance that helps increase the anti-inflammatory, antioxidant, and anti-tumor effects of *Dendrobium officinale* juices [12]. Kusnezoff Monkshood root has high toxicity, and improper use can easily lead to poisoning and life-threatening effects. Fungal fermentation of Kusnezoff Monkshood root can significantly reduce the contents of aconitine, mesaconitine, and hypaconitine, which are highly toxic ingredients [13].

The chemical composition of traditional Chinese medicines (TCMs) is of great significance in elucidating the action mechanism of TCMs and guiding the safe clinical use of medicines. Currently, the quality standard of fermented GTHP is not optimal. Only the ultraviolet spectrophotometer method is utilized to determine the contents of polysaccharides and peptides. Some compounds may co-absorb with the target compounds, thereby affecting the results. Indicator components and exclusive compounds for content determination are lacking, which may lead to forgeries or affect product quality. In recent years, ultra-high-performance liquid chromatography-quadrupole-Orbitrap high-resolution mass spectrometry (UHPLC-Q-Orbitrap HRMS) technology has been utilized in the fields of medicine and food. This technology can identify specific ions by comparing their characteristic secondary mass spectra, enabling fast, simple, and accurate qualitative analysis. The technology can allow for analyzing complex TCMs, identifying active ingredients, and detecting toxic components. High-performance liquid chromatography (HPLC) can achieve high separation efficiency, excellent detection sensitivity, good reproducibility, high quantitative accuracy, and a wide range of applications through mobile phase optimization.

Currently, studies on the research regarding the chemical composition of GTHP are few. In the present study, a rapid and reliable multicomponent determination method, specifically an HPLC method combined with fingerprint analysis, was developed for the quality evaluation of GTHP after fermentation. To the best of our knowledge, this is the first report on the analysis of the chemical composition of GTHP through the UHPLC-Q-Orbitrap HRMS technique. This study provides an important foundation for the quality control of GTHP and its potential clinical application.

## 2. Results

### 2.1. Results of UHPLC-Q-Orbitrap HRMS Analysis

**Figure 1:**
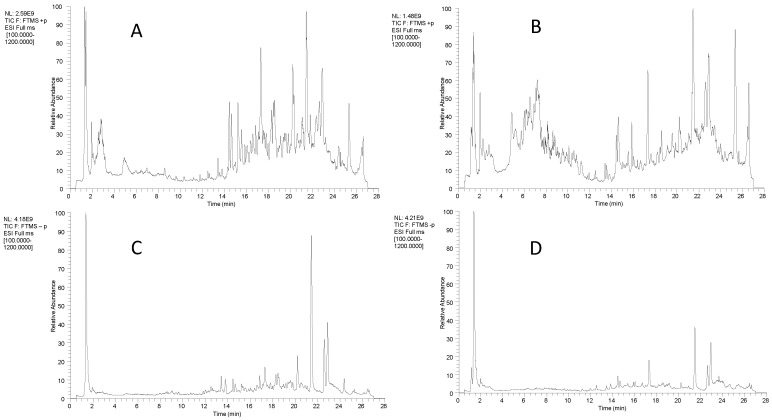
Positive and negative ion chromatograms of GTHP before and after fermentation. Note: Positive ion mode total ion flow plots before (**A**) and after (**B**) GTHP fermentation. Negative ion mode total ion flow plots before (**C**) and after (**D**) GTHP fermentation.

Comparison of positive and negative total ion flow plots of GTHP before and after fermentation can illustrate the changes in chemical composition resulting from fermentation. The results are depicted in Figure 1. The initial attribution of each component was determined, and compositional identification was conducted in conjunction with reference substances and relevant literature. Seventy-nine compounds were identified, including thirteen flavonoids, twelve organic acids, eight nucleosides, eight terpenoids, four amides, four esters, three alkaloids, three steroids, two amino acids, two furfurals, two sphingolipids, two vitamins, and sixteen others. The results are shown in Table 1. GTHP mainly consisted of flavonoids, organic acids, nucleosides, and terpenoids, which were analyzed separately as examples.

#### 2.1.1. Flavonoid Compounds of GTHP

**Figure 2:**
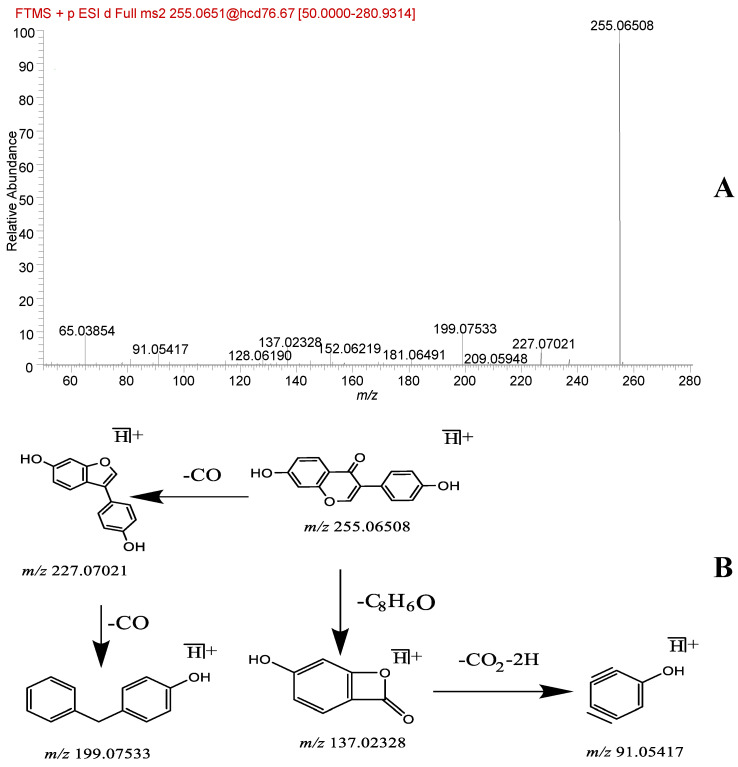
Daidzein fragmentation spectra (**A**) and cleavage pathway (**B**).

**Figure 3:**
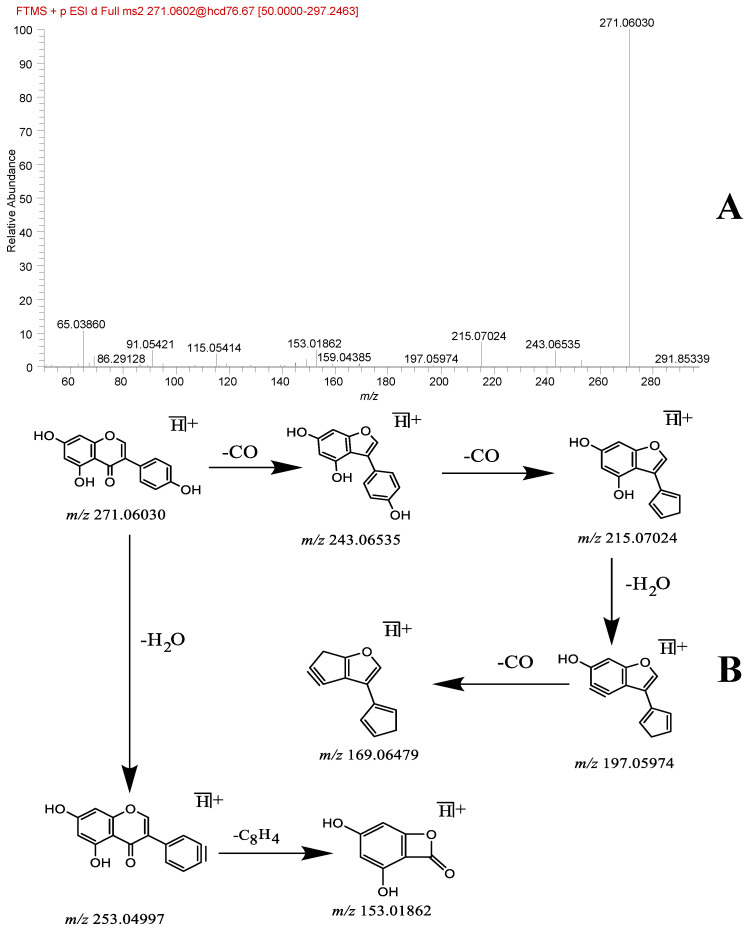
Genistein fragmentation spectra (**A**) and cleavage pathway (**B**).

A total of 13 flavonoid analogs were identified, namely gaidzin, glycitin, puerarin, genistin, daidzein, chrysin, glycitein, 4’,7-dihydroxyflavanone, genistein, naringenin, fisetin, kaempferide, and ar-Turmerone. Compounds such as daidzein (**42**) and genistein (**46**) were used as examples. The primary mass spectrum showed daidzein *m/z* 255.06508 [M + H]^+^ quasi-molecular ion peaks with the chemical formula C_15_H_10_O_4_. The secondary fragmentation ion information includes *m/z* 227.07021 [M + H − CO]^+^, *m/z* 199.07533 [M + H − 2CO]^+^, *m/z* 137.02328 [M + H − C_8_H_6_O]^+^, and *m/z* 91.05417 [M + H − C_8_H_6_O − CO_2_ − 2H]^+^. This fragmentation pattern is similar to the cleavage pathway reported in the literature [18]. The primary mass spectrum of genistein yielded a quasi-molecular ion peak *m/z* 271.0603 [M + H]^+^ with a chemical formula of C_15_H_10_O_5_. Secondary fragment ions were present at *m/z* 253.04997 [M + H − H_2_O]^+^, 243.06535 [M + H − CO]^+^, 215.07024 [M + H − 2CO]^+^, 197.05974 [M + H − 2CO − H_2_O]^+^, 169.06479 [M + H − 3CO − H_2_O]^+^, and 153.01862 [M + H − H_2_O − C_8_H_4_]^+^. Similar cleavage pathways were reported in a previous study [19]. Moreover, the compounds were finally identified as daidzein and genistein through a comparison of the primary and secondary mass spectrometry cleavage fragments of the control with the extracted ion peak positions and relative retention times. The corresponding mass spectrometry cleavage pathways are shown in Figure 2 and Figure 3.

#### 2.1.2. Organic Acids and Nucleoside Compounds in GTHP

**Figure 4:**
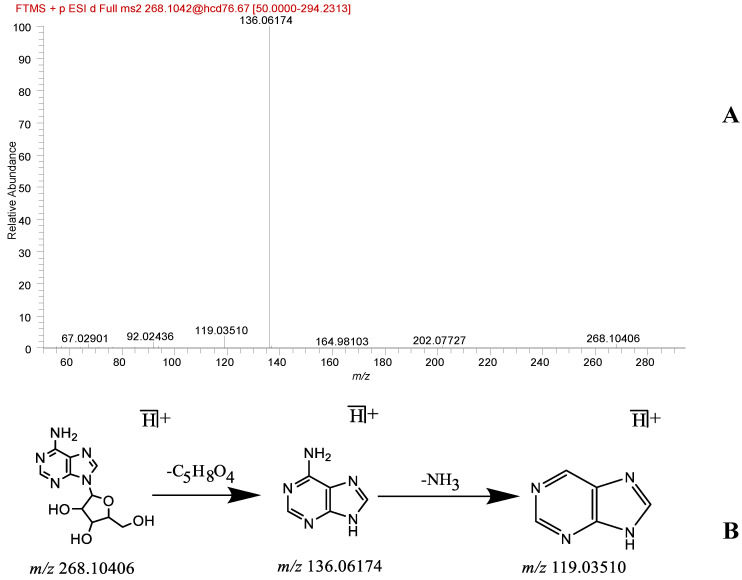
Adenosine fragmentation spectra (**A**) and cleavage pathway (**B**).

Twelve organic acid-like and eight nucleoside-like components were identified in this experiment. The organic acid compounds included methylsuccinic acid, pantothenic acid, 3,4-dihydroxyphenylacetic acid, 3-hydroxy-3-methylbutanoic acid, 5-hydroxyindole-3-acetic acid, terephthalic acid, benzoic acid, mesaconic acid, linoleic acid, palmitoleic acid, palmitic acid, and oleic acid. The nucleoside analogs comprised cytidine, cytosine, 2,6-dihydroxypurine, uridine, adenosine, guanine, guanosine, and uracil. Compounds such as linoleic acid (**71**) and adenosine (**12**) were used as examples. The presence of linoleic acid, with the chemical formula C_18_H_32_O_2_, at the *m/z* 279.23306 [M − H]^−^ quasi-molecular ion peak was identified in the primary mass spectrum. The secondary fragmentation ion information was *m/z* 261.22192 [M − H − H_2_O]^−^, which was consistent with the cleavage pathway reported in the literature [24]. The primary mass spectrum of adenosine yielded a quasi-molecular ion peak at *m/z* 268.10406 [M + H]^+^ with the chemical formula C_10_H_13_N_5_O_4_. Secondary fragmentation ion information included *m/z* 136.06174 [M + H − C_5_H_9_O_4_]^+^ and 119.03510 [M + H − NH_3_]^+^, which aligns with the cleavage pathway reported in the literature [16]. The compound was finally identified as adenosine through a comparison of the primary and secondary mass spectrometry cleavage fragments of the control with the extracted ion peak positions and relative retention times. The corresponding mass spectrometry cleavage pathway is shown in Figure 4.

#### 2.1.3. Terpenoid Compounds in GTHP

Eight terpenoid analogs were identified, namely, armillarinin, soyasaponin I, armillarilin, armillarin, dehydroeburicoic acid, armillaribin, armillaricin, and melleolide. Compound (**61**), armillaricin, was used as an example. The presence of armillaricin with a quasi-molecular ion peak at *m/z* 431.16202 [M + H]^+^ and a chemical formula of C_24_H_27_O_5_Cl was identified in the primary mass spectrum. In addition, secondary fragment ion information showed peaks at *m/z* 215.14302 [M + H − C_9_H_8_O_4_Cl]^+^, 199.01567 [M + H − C_15_H_20_O_2_]^+^, and 187.14812 [M + H − C_9_H_8_O_4_Cl − CO]^+^. These findings align with the cleavage pathway reported in the literature [22].

### 2.2. Orthogonal Projections to Latent Structures Discriminant Analysis of Pre- and Post-Fermented GTHP

**Figure 5:**
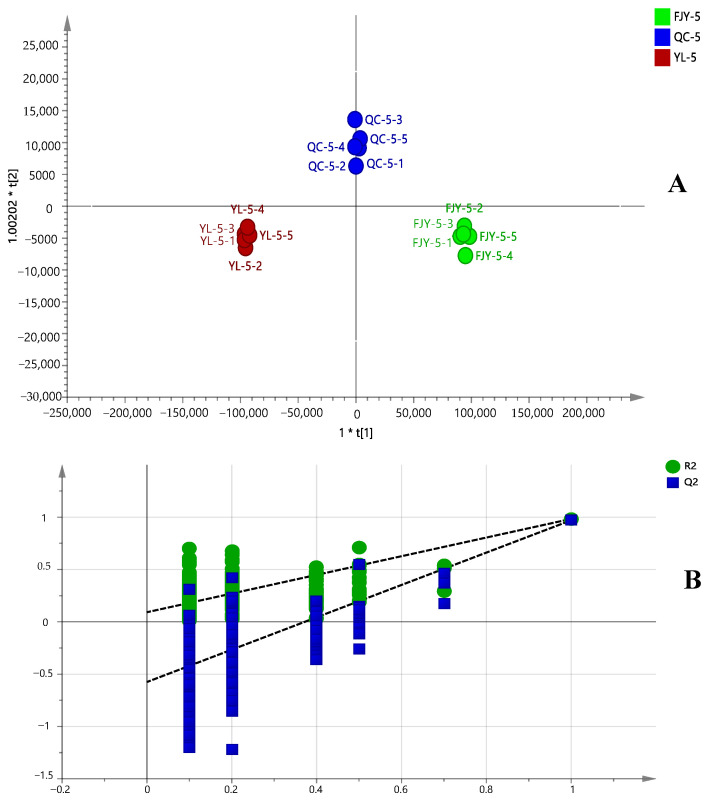
OPLS-DA analysis (**A**) and model replacement test (**B**) of pre- and post-fermented GTHP. Note: “FJY-5” indicates that the fermented samples were extracted five times. “YL-5” indicates that the samples before fermentation were extracted five times. “QC-5” denotes a set of five quality control samples taken before and after fermentation. YL-5-1–5, FJH-5-1–5, and QC-5-1–5 represent the injection numbers of the five samples taken before fermentation, after fermentation, and for quality control purposes, respectively.

**Figure 6:**
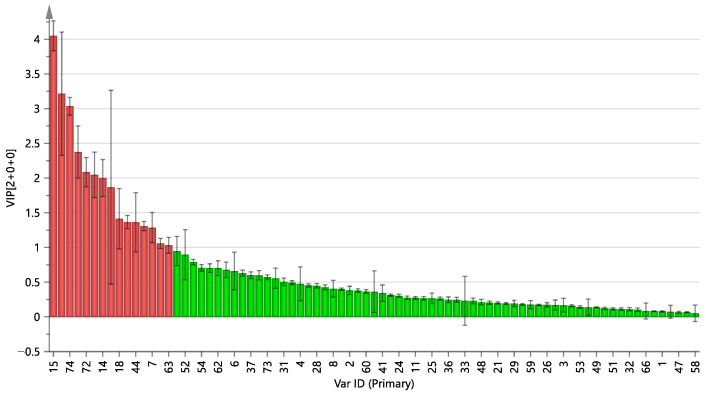
Plot of VIP values of GTHP and QC before and after fermentation. Note: Red indicates VIP value > 1; green indicates VIP value < 1.

To identify the marker compounds that distinguish the differences between GTHP before and after fermentation, five portions from each of pre- and post-fermented GTHP were extracted by preparing test solutions according to the method outlined in Section 4.2.1. Subsequently, quality control (QC) solutions were prepared. The samples were analyzed according to the conditions specified in Section 4.3.1, via UHPLC-Q-Orbitrap HRMS. The raw data from tandem mass spectrometry (MSE) were processed for alignment, deconvolution, and data reduction using Xcalibur 4.5, which detects chromatographic peaks to extract variables (tR, *m*/*z*, and intensity), normalize, and align similar variables to create a data matrix before presenting the results in a marker table. An Xcalibur 4.5 processing method was developed using the following main parameters: retention time range, 0–28 min; minimum intensity, 5%; mass range, 100–1200 Da; marker intensity threshold, 2000 counts. All processed data, including the *m*/*z*-tR pairs from each data file and the corresponding intensities of all the detected peaks, were exported and analyzed using the SIMCA 14.1 software. In different samples, components with the same tR and *m*/*z* values were considered identical. Orthogonal projections to latent structures discriminant analysis (OPLS-DA) was conducted to achieve maximum separation between two distinct samples and the potential chemical markers responsible for the differences. In the sufficient permutation test, the R2X, R2Y, and Q2 of the OPLS-DA model were 0.986, 0.970, and 0.946, respectively, indicating acceptable validity for the subsequent identification of the characteristic markers (Figure 5A). To prevent overfitting and maintain the accuracy of the results, the established OPLS-DA model was internally validated using the 200-substitution test model. The results are depicted in Figure 5B. The vertical coordinates of R2 and Q2 in the upper right corner are higher than those of the leftmost R2 and Q2. The slopes for R2 and Q2 are 0.0919 and −0.575, respectively. In addition, the blue regression line at point Q2 intersects the vertical axis on the left at a point below zero. This indicates that the constructed model is reliable, there is no overfitting phenomenon, and the results are dependable for use in marker screening [27]. The variable importance for the projection (VIP) value indicates that the greater the value of the weights, the greater the ability to differentiate between the samples. VIP value is shown in Figure 6. When VIP value > 1, the difference in ingredients includes uracil, daidzein, ergosterol, adenosine, oleamide, genistein, guanosine, 5-hydroxymethylfurfural, indoline, oleic acid, glycitein, genistin, 2,6-Dihydroxypurine, daidzin, coriolic acid, and cycluron.

### 2.3. Establishment of Fingerprinting and Multicomponent Content Determination of GTHP

#### 2.3.1. Establishment of Fingerprints for GTHP

**Figure 7:**
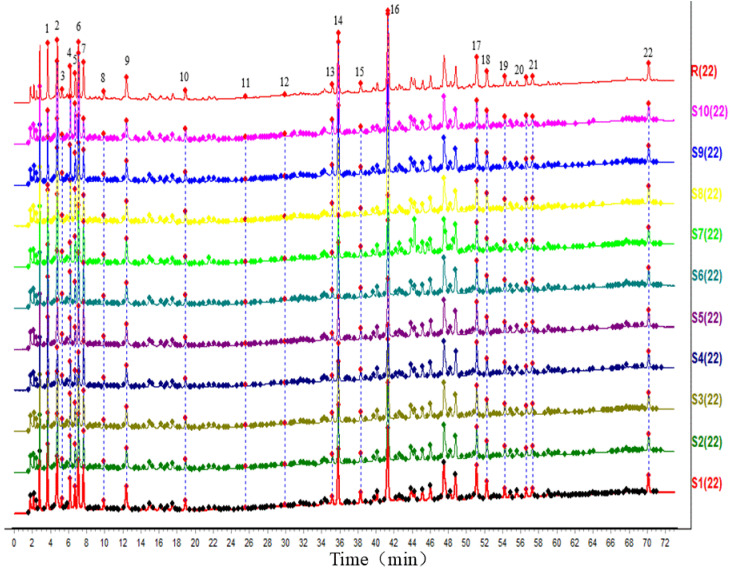
Ten batches of fermented GTHP samples. Note: S1–S7 refer to GTHP fermented by Luoyang Wokang Pharmaceutical Co. S8–S10 refer to GTHP fermented by Jiangsu Shenhua Pharmaceutical Co. R represents the control spectrum generated by 22 identified peaks.

**Figure 8:**
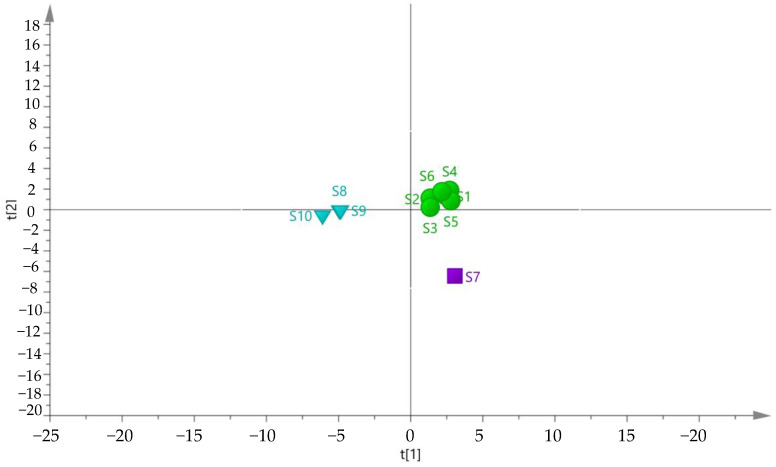
PCA plot of 10 batches of GTHP after fermentation.

**Figure 9:**
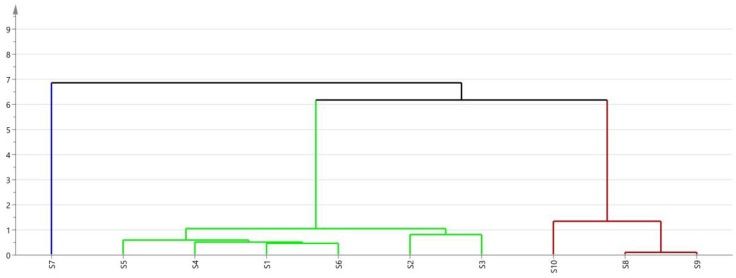
CA plot of 10 batches of GTHP after fermentation.

Ten batches of fermented GTHP samples (S1–S10) were prepared as solutions according to the method outlined in Section 4.2.2 and then injected for analysis as outlined in Section 4.3.2. The chromatograms were imported into the “Traditional Chinese Medicine Chromatographic Fingerprint Similarity Evaluation System (2012 version)”. The chromatogram of sample S1 was used as the reference, and multi-point correction was performed to generate superimposed chromatograms and the control chromatograms (R). Twenty-two peaks were identified, and the results are shown in Figure 7. The peak areas of the plots were subjected to principal component analysis (PCA) and cluster analysis (CA) using SIMCA 14.1 software. The results are depicted in Figure 8 and Figure 9, respectively. In the PCA analysis, *R2X* and *Q2* values in PCA were 0.968 and 0.856, respectively. The PCA and CA can be utilized to categorize the ten batches of GTHP into three groups. Samples S1–S6 belong to the first category, S7 belongs to the second category, and samples S8–S10 belong to the third category. Samples S1–S7 were obtained from the same manufacturer, and they formed a distinct cluster, possibly influenced by seasonal fermentation. These findings indicate variations between GTHP from different manufacturers and batches. The results of similarity evaluation are presented in Table 2. The similarity between the samples of each batch and the control atlas was ≥0.971, indicating minimal differences in the quality of samples from different batches and manufacturers.

#### 2.3.2. Linear Investigation Results

The regression equations for uracil, guanosine, adenosine, 5-HMF, daidzin, genistin, glycitein, daidzein, genistein, and ergosterol were derived by plotting the standard curve with the concentration (X) in µg/mL as the horizontal coordinate and the peak area (Y) as the vertical coordinate. The results are shown in Table 3.

#### 2.3.3. Methodological Investigations

The results of the precision test showed that the relative standard deviations (RSDs) of the peak areas of uracil, guanosine, adenosine, 5-HMF, daidzin, genistin, glycitein, daidzein, genistein, and ergosterol were 0.69%, 0.28%, 0.32%, 0.53%, 0.40%, 0.24%, 0.44%, 0.25%, 0.70%, and 0.49%, respectively. These values indicate good precision of the experimental apparatus. The stability test results showed that the RSDs of the peak areas of uracil, guanosine, adenosine, 5-HMF, daidzin, genistin, glycitein, daidzein, genistein, and ergosterol were 0.75%, 1.17%, 0.84%, 1.04%, 1.53%, 1.51%, 1.07%, 0.60%, 0.54%, and 0.72%, respectively, indicating that the solution was stable for over 12 h. The results of the repeatability test experiments also demonstrated that the peak area RSDs of uracil, guanosine, adenosine, 5-HMF, daidzin, genistin, glycitein, daidzein, genistein, and ergosterol were 0.69%, 0.84%, 0.92%, 0.90%, 1.77%, 1.35%, 1.26%, 0.42%, 0.83%, and 0.88%, respectively, confirming the reproducibility of the experimental method. The results of sample spiking recovery tests showed that the average spiking recoveries of uracil, guanosine, adenosine, 5-HMF, daidzin, genistin, glycitein, daidzein, genistein, and ergosterol were 99.32%, 98.06%, 98.75%, 98.25%, 98.56%, 97.45%, 98.26%, 99.11%, 99.08%, and 99.65%, respectively. The RSDs were 0.74%, 1.04%, 0.90%, 1.55%, 1.86%, 1.65%, 1.53%, 1.05%, 0.92%, and 1.30%, respectively. These results demonstrate the accuracy of the experimental methodology. The specific results are presented in Table 4. The limits of detection (LODs) for uracil, guanosine, adenosine, 5-HMF, daidzin, genistin, glycitein, daidzein, genistein, and ergosterol were 0.25 μg/mL, 0.22 μg/mL, 0.37 μg/mL, 0.33 μg/mL, 0.07 μg/mL, 0.07 μg/mL, 0.07 μg/mL, 0.21 μg/mL, 0.09 μg/mL, and 0.15 μg/mL, respectively, and the limits of quantification (LOQs) were 0.84 μg/mL, 0.73 μg/mL, 1.23 μg/mL, 1.10 μg/mL, 0.23 μg/mL, 0.22 μg/mL, 0.22 μg/mL, 0.71 μg/mL, 0.28 μg/mL, and 0.51 μg/mL, respectively. The method displayed high detection sensitivity, and the analytical conditions could be met.

#### 2.3.4. Content Analysis of GTHP Samples before and after Fermentation

**Figure 10:**
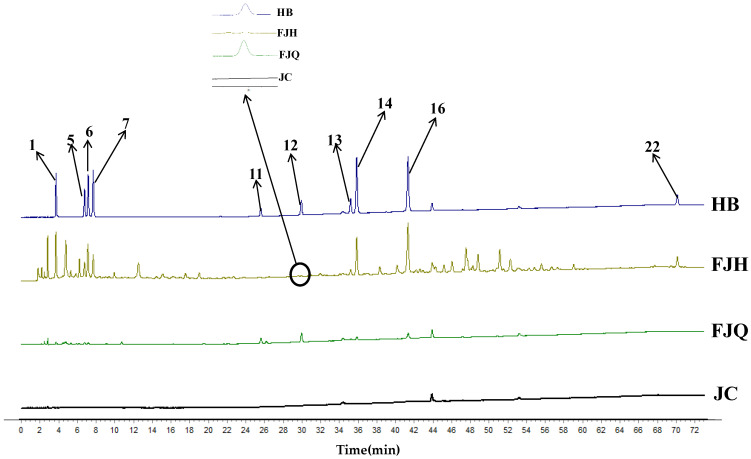
HPLC profiles of GTHP samples before and after fermentation. Note: “JC” blank solution; “FJQ” pre-fermentation GTHP solution; “FJH” post-fermentation GTHP solution; “HB” mixed reference solution; uracil (**1**), guanosine (**5**), adenosine (**6**), 5-HMF (**7**), daidzin (**11**), genistin (**12**), glycitein (**13**), daidzein (**14**), genistein (**16**) and ergosterol (**22**) are consistent with the fingerprinted peak numbers.

**Figure 11:**
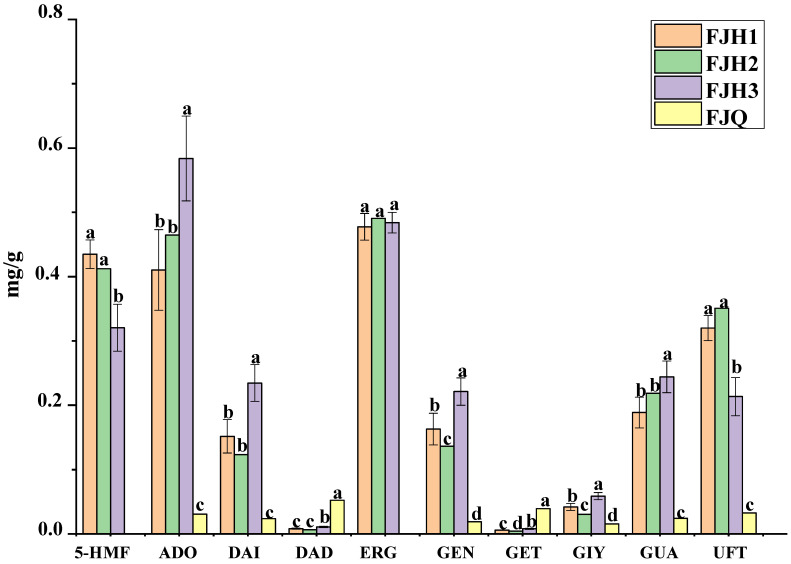
The results of the ten target compounds in GTHP were analyzed using SPSS 26.0 and Origin 2018 software. Note: Different letters for the same component indicate significant differences at *p* < 0.05. 5-hydroxymethylfurfural (5-HMF), adenosine (ADO), daidzein (DAI), daidzin (DAD), ergosterol (ERG), genistein (GEN), genistin (GET), glycitein (GIY), guanosine (GUA), and uracil (UFT).

GTHP before (S11) and after (S1) fermentation and the solution to be tested were extracted according to the method outlined in Section 4.2.2. In addition, a mixed control solution was prepared according to the method outlined in Section 4.2.3. Subsequently, the samples were analyzed via injection under the conditions outlined in Section 4.3.2. The results are shown in Figure 10. Samples S2–S10 were prepared and analyzed via injection in the same manner. The concentrations of uracil, guanosine, adenosine, 5-HMF, daidzin, genistin, glycitein, daidzein, genistein, and ergosterol in each sample were determined through the regression equation described in Section 2.3.2. The results are presented in Table 5. In Section 2.3.1, the fermented GTHP was categorized into three groups: S1–S6 for category 1 (FJH1), S7 for category 2 (FJH2), and S8–S10 (FJH3) for category 3. In addition, the pre-fermented S11 (FJQ) was categorized as category 4. The concentrations of the four categories were analyzed using SPSS data and plotted in Origin (Figure 11). After fermentation, 10 chemical constituents in GTHP underwent changes. The amounts of uracil, guanosine, adenosine, 5-HMF, glycitein, daidzein, genistein, and ergosterol increased, while the amounts of daidzin and genistin decreased. Among them, 5-HMF and ergosterol were possibly new components produced by the fermentation process, with contents exceeding 0.4348 mg/g and 0.4775 mg/g, respectively. This indicates that the components were interconverted during the fermentation process, or other substances may have been synthesized from raw materials or through oxidative degradation to produce other substances.

## 3. Discussion

According to Figure 11, the levels of uracil, guanosine, adenosine, 5-HMF, glycitein, daidzein, genistein, and ergosterol in GTHP increased after fermentation, while the levels of daidzin and genistin decreased. This suggests the possibility of mutual transformation between compounds during the fermentation process, the synthesis of other substances, or the oxidative degradation of certain substances into different compounds. The levels of 5-HMF, adenosine, daidzein, genistein, and uracil in GTHP showed significant variation after fermentation, indicating that manufacturers should monitor these changes during production to assess the product’s quality.

According to the UHPLC-Q-Orbitrap HRMS technology, organic acids, including linoleic acid, palmitoleic acid, palmitic acid, and oleic acid, were detected in GTHP. These organic acids may contribute to the aroma of the powder. In addition, ergosterol was observed after fermentation, which may be a result of the breakdown of certain substances. The genistein content in GTHP increased during fermentation, indicating that the initial genistin present before fermentation may be utilized as the fermentation advances. According to several studies, ergosterol has been found to have beneficial effects in resisting Alzheimer’s disease, diabetes, fatty liver, and providing neuroprotective effects [28]. Adenosine has been shown to maintain the stability of the nervous system, regulate vascular activity, promote sleep, facilitate retinal neovascularization, and exhibit antiarrhythmic effects [29,30]. In addition, 5-HMF has been found to protect nerves, improve Alzheimer’s disease, and have anti-hypoxic effects [31,32]. However, it is important to consider the potential toxicity of 5-HMF, which primarily causes nephrotoxicity through oxidative stress, energy metabolism disorders, purine metabolism disorders, and amino acid metabolism imbalances. Although specific limits are not specified, a glucose content above 0.02% should alert the manufacturer [33]. Uracil has been found to possess bactericidal, antiviral, and anti-tumor effects [34,35]. Guanosine has been shown to resist epileptic seizures and relax the aorta [36,37]. Daidzein has been found to improve ischemic brain damage, cerebral edema, and endothelial dysfunction, and exhibit anti-epileptic effects [38]. Last, genistein has been found to exhibit anti-Aβ neurotoxicity, regulate blood sugar and blood lipids, and play a role in atherosclerosis [39]. Combining VIP > 1, functional indication analysis, and high-content chemical components as QC indicators, ergosterol, adenosine, 5-HMF, uracil, guanosine, daidzein, and genistein may serve as the main markers of GTHP. These markers provide valuable information for establishing quality evaluation.

This paper presents the first systematic study of the chemical composition of GTHP before and after fermentation through UHPLC-Q-Orbitrap HRMS analysis. Mass spectrometry data of the characteristic components are provided, and the experimental results serve as the foundation for the analysis of the blood components and pharmacokinetics of GTHP. Fingerprints and multicomponent compound content determination of GTHP after fermentation were established via HPLC to elucidate the overall quality characteristics and differences of GTHP. The paper provides a scientific basis for the QC and clinical application of GTHP.

## 4. Materials and Methods

### 4.1. Materials

#### 4.1.1. Experimental

An Ultimate 3000-Orbitrap Exploris 240 liquid mass spectrometer (Thermo Fisher Scientific, MA, USA) and a Shimadzu LC-20AD high-performance liquid chromatograph (Shimadzu, Kyoto, Japan) were used in the study. In addition, a BSA224S-CW 1/10,000 balance and a BT25S 1/100,000 balance (Sartorius Technology Instrument Co., Ltd., Gottingen Germany), a UPT-II-10T ultrapure water device (Chengdu Ultrapure Technology Co., Ltd., Chengdu, China), and a KQ-500DV ultrasonic cleaner (Kunshan Ultrasonic Instrument Co., Ltd., Kunshan, China) were utilized.

#### 4.1.2. Experimental Reagents and Medicinal Materials

Following the fermentation process, there were 10 batches of GTHP, with 7 batches provided by Luoyang Wokang Pharmaceutical Co. The batch numbers were 220101 (S1), 220102 (S2), 220103 (S3), 220201 (S4), 220202 (S5), 220203 (S6), and 220701 (S7). Another three batches with the numbers 211108 (S8), 211109 (S9), and 211110 (S10) were provided by Jiangsu Shenhua Pharmaceutical Co. Pre-fermented GTHP from Luoyang Wokang Pharmaceutical Co., batch number 20211202 (S11), was used. The water used was homemade double-distilled water from the laboratory. Pure acetonitrile and formic acid were used for mass spectrometry (both from Thermo Fisher Scientific (China) Co., Shanghai, China), while pure methanol and acetic acid were used for chromatography (Anhui Tiandi High Purity Solvent Co., Ltd., Anhui, China). The remaining reagents were of analytical purity. The control products are listed in Table 6, and their purities are ≥98%.

### 4.2. Methods

#### 4.2.1. Preparation of HRMS Test Solution

Each GTHP was weighed precisely to 1.0 g and mixed with 10 mL of methanol (HPLC grade). Each sample was extracted at 30 °C for 60 min in an ultrasonic bath (power: 500 W; frequency: 40 kHz). After cooling to 20 °C, the weight loss was replenished with methanol. Then, 1 mL filtrate of the extract was transferred to a 2 mL centrifuge tube and evaporated to dryness using a high-speed cryo-centrifuge for ~3 h. Afterward, 500 μL of methanol (MS) was added to re-dissolve the extract, and the mixture was centrifuged in a high-speed centrifuge at 12,000 r/min for 10 min. The supernatant was then filtered using a 0.22 µm syringe filter and injected into the UHPLC system.

#### 4.2.2. Preparation of HPLC Test Solutions

Each GTHP was weighed precisely (1.0 g) and mixed with 10 mL of methanol (HPLC grade). Each sample was extracted at 30 °C for 60 min in an ultrasonic bath (power: 500 W; frequency: 40 kHz). After cooling to 20 °C, the weight loss was replenished with methanol. The extraction solution was then filtered using a syringe filter (0.22 µm) and injected into the HPLC system.

#### 4.2.3. Preparation of Standard Solutions and Standard Curves for 10 Chemical Components

Appropriate amounts of uracil, guanosine, adenosine, 5-HMF, daidzin, genistin, glycitein, daidzein, genistein, and ergosterol reference substances were accurately weighed. Methanol was then added to prepare the mixed reference solution containing the following concentrations: 32.84, 34.31, 58.82, 74.12, 10.15, 8.92, 13.24, 36.03, 25.29, and 63.73 µg/mL, respectively.

### 4.3. Analysis Conditions

#### 4.3.1. UHPLC and Mass Spectrometry Conditions

An Ultimate 3000-Orbitrap Exploris 240 liquid mass spectrometer (Thermo Fisher Scientific, USA) with a Hypersil GOLD column (100 × 2.1 mm, 1.9 μm, Thermo Fisher Scientific, USA) was used. The flow rate was 0.3 mL/min, the column temperature was 35 °C, and the mobile phase consisted of acetonitrile (A) and 0.1% formic acid (*v*/*v*, B). The run time was 28 min. The elution process was carried out using gradients of solvent A and B. The gradient elution program was used as follows: 0–1 min, 2% A; 1–8 min, 2–20% A; 8–14 min, 20–70% A; 14–22 min, 70–95% A; 22–24 min, 95% A; 24–24.5 min, 95–2% A; 24.5–28 min, 2% A. The sample size was 2 μL.

The scanning range for positive and negative ion detection modes was *m/z* 100–1200. The positive and negative ion spray voltages were 3.5 and −3.0 kV, respectively. The sheath gas flow rate was 25 arbitrary units (arb), the auxiliary gas flow rate was 10 arb, and the auxiliary temperature was 350 °C. The ion transfer tube temperature was 350 °C.

#### 4.3.2. HPLC Fingerprinting and Multicomponent Content Determination Conditions

The analysis was performed on a Waters Symmetry C_18_ column (4.6 mm × 250 mm, 5 μm). The mobile phase consisted of solution A (methanol) and solution B (0.1% acetic acid aqueous solution). The run time was 73 min. The elution process was carried out using gradients of solvent A and B. The gradient elution program was used as follows: 0–20 min, 5–35% A; 20–28 min, 35–55% A; 28–40 min, 55–75% A; 40–65 min, 75–100% A; 65–73 min, 100% A. The injection volume was 3 μL. The column temperature was maintained at 30 °C. The flow rate was 1 mL/min. The diode array detection wavelength was 254 nm.

### 4.4. Investigation of Linear Relationship

A total of 30, 100, 200, 400, 600, 800, and 1000 μL of mixed standard solutions were each placed in a 1 mL volumetric flask. Methanol solution was then added to each volumetric flask. Subsequently, the standard solutions were injected into the HPLC system and analyzed via the chromatographic method. The calibration curves were plotted with the concentration (µg/mL) on the abscissa (*X*) and the mean peak area on the ordinate (*Y*).

#### 4.4.1. Precision Test

The powder from the S1 sample was prepared in a test solution following the procedure outlined in Section 4.2.2. The sample was injected into the HPLC system six times consecutively under the analytical conditions specified in Section 4.3.2. The RSD values of the peak areas of uracil, guanosine, adenosine, 5-HMF, daidzin, genistin, glycitein, daidzein, genistein, and ergosterol were calculated.

#### 4.4.2. Stability Test

The powder from the S1 sample was prepared in a test solution following the method outlined in Section 4.2.2. The samples were injected into the HPLC system after 0, 2, 4, 6, 8, and 12 h according to the analytical conditions specified in Section 4.3.2. The RSD values of the peak areas for uracil, guanosine, adenosine, 5-HMF, daidzin, genistin, glycitein, daidzein, genistein, and ergosterol were calculated.

#### 4.4.3. Repeatability Test

The S1 sample powder was divided into six portions to prepare a test solution according to the method outlined in Section 4.2.2. Then, the sample was injected into the HPLC system according to the analytical conditions specified in Section 4.3.2. The RSD of the peak areas of uracil, guanosine, adenosine, 5-HMF, daidzin, genistin, glycitein, daidzein, genistein, and ergosterol were calculated.

#### 4.4.4. Sample Addition Recovery Test

The powder of the GTHP sample (S7) with known content was accurately weighed into six parts, each approximately 0.5 g, and an appropriate amount of mixed control solution was added, respectively, ensuring a ratio of approximately 1:1 (*w*/*w*) between the original amount and the amount added. The samples were then prepared according to the method outlined in Section 4.2.2. Subsequently, the test solution was injected into the HPLC system according to the analysis conditions specified in Section 4.3.1. Mean recoveries and RSD values for uracil, guanosine, adenosine, 5-HMF, daidzin, genistin, glycitein, daidzein, genistein, and ergosterol were calculated.

#### 4.4.5. LOD and LOQ Tests

The powder of sample S1 was accurately weighed, and an appropriate amount of powder was prepared in the test solution according to the method outlined in Section 4.2.2. The sample was injected into the HPLC system 10 times consecutively under the analytical conditions of Section 4.3.2. The LOD and LOQ of the method were determined to be 3 and 10 times the signal-to-noise ratios, respectively.

### 4.5. Data Analysis and Processing

#### 4.5.1. Data Processing UHPLC-Q-Orbitrap HRMS Chromatograms

All UHPLC-Q-Orbitrap HRMS data were extracted and processed on an Xcalibur 4.5 workstation using the type of adduct ion peak (negative ion mode, [M − H]^−^ and [M − Na]^−^; in positive ion mode, [M + H]^+^ and [M + Na]^+^). When matching with databases such as mzCloud and mzVault, compounds were attributed and identified based on retention time, quasi-molecular ion peaks from primary mass spectra, and characteristic fragment ion information from secondary mass spectra. Comprehensive references, controls, ChemSpider, PubChem, and SciFinder databases were used to accurately compare and validate the identified components.

#### 4.5.2. Processing of GTHP Sample Content Determination Data

Sample powders S1–S11 were obtained and processed following the procedure outlined in Section 4.2.2 to produce a test solution. Subsequently, the solution was injected into the HPLC system and analyzed according to the analysis conditions detailed in Section 4.3.2. The peak areas of uracil, guanosine, adenosine, 5-HMF, daidzin, genistin, glycitein, daidzein, genistein, and ergosterol. were measured. The quantities of each index component in the samples were determined using a regression equation.

#### 4.5.3. SPSS Software Processing of Pre- and Post-Fermentation GTHP Data

Through PCA and CA, samples S1–S10 were categorized into three classes. S11 was added, resulting in a total of four classes. The mean and variance results for each category were calculated using SPSS software and then imported into Origin for graphing.

## Figures and Tables

**Table 1:** UHPLC-Q-Orbitrap HRMS-based identification of compounds in GTHP before and after fermentation.

Serial Number	Sort	Compound	Retention Time	Molecular Formula	Ion Mode	Calculated Value (*m/z*)	Measured Value (*m/z*)	Error Value/×10^−6^	Fragmentation	Identification Method	FJH	FJQ
1	Alkaloid	Choline	1.29	C_5_H_13_NO	[M + H]^+^	104.10699	104.10683	−1.54	60.08076, 58.06512	Database	+	+
2	Alkaloid	Trigonelline	1.38	C_7_H_7_NO_2_	[M + H]^+^	138.05496	138.0549	−0.40	110.05998, 94.06508	Database	+	+
3	Nucleoside	Cytidine *	1.41	C_9_H_13_N_3_O_5_	[M + H]^+^	244.09279	244.09276	−0.12	112.05054	Database, literature [14]	+	+
4	Nucleoside	Cytosine	1.44	C_4_H_5_N_3_O	[M + H]^+^	112.05054	112.05040	−1.24	95.02391, 94.03985, 69.04473	Database, literature [14]	+	+
5	Alkaloid	Betaine *	1.88	C_5_H_11_NO_2_	[M + H]^+^	118.08626	118.08625	−0.08	58.06511, 59.07307	Database	+	+
6	Vitamin	Nicotinic acid *	1.94	C_6_H_5_NO_2_	[M + H]^+^	124.03931	124.03925	−0.44	80.04941, 96.04428, 78.03378	Database	+	+
7	Nucleoside	2,6-Dihydroxypurine *	1.99	C_5_H_4_N_4_O_2_	[M − H]^−^	151.02615	151.02625	0.66	108.02042	Database, literature [15]	+	+
8	Nucleoside	Uridine *	2.00	C_9_H_12_N_2_O_6_	[M + H]^+^	245.07681	245.07684	0.12	113.03448	Database, literature [14]	+	+
9	Other	Fumaric acid	2.03	C_4_H_4_O_4_	[M − H]^−^	115.00368	115.00382	1.20	71.01396	Database	+	−
10	Other	2-Furoic acid	2.09	C_5_H_4_O_3_	[M − H]^−^	111.00877	111.00905	2.55	67.01904	Database	+	+
11	Other	2-Deoxypentose	2.20	C_5_H_10_O_4_	[M − H]^−^	133.05063	133.05081	1.34	115.00375, 71.01393	Database	+	−
12	Nucleoside	Adenosine *	2.44	C_10_H_13_N_5_O_4_	[M + H]^+^	268.10403	268.10406	0.11	136.06174, 119.03510	Database, literature [14,16]	+	+
13	Nucleoside	Guanine	2.49	C_5_H_5_N_5_O	[M + H]^+^	152.05669	152.05663	−0.37	135.03009, 128.04570, 110.03478	Database	+	+
14	Nucleoside	Guanosine *	2.60	C_10_H_13_N_5_O_5_	[M + H]^+^	284.09894	284.09885	−0.32	152.05670	Database, literature [14]	+	+
15	Nucleoside	Uracil *	2.64	C_4_H_4_N_2_O_2_	[M + H]^+^	113.03455	113.03448	−0.62	96.00786	Database, literature [14]	+	+
16	Vitamin	Ascorbic acid *	3.30	C_6_H_8_O_6_	[M − H]^−^	175.02481	175.0253	2.79	115.00316, 71.01390	Database	+	−
17	Furfurals	5-Methyl-2-furaldehyde *	4.63	C_6_H_6_O_2_	[M + H]^+^	111.04406	111.04408	0.22	83.04917, 55.05423	Database	+	−
18	Other	Indoline	4.81	C_8_H_9_N	[M + H]^+^	120.08078	120.08072	−0.47	103.05418, 93.06983	Database	+	+
19	Furfurals	5-hydroxymethylfurfural *	4.91	C_6_H_6_O_3_	[M + H]^+^	127.03898	127.03900	0.23	109.02830	Database, literature [17]	+	−
20	Amino acids	D-phenylalanine *	5.04	C_9_H_11_NO_2_	[M − H]^−^	164.07170	164.07182	0.73	147.04529	Database	+	+
21	Amino acids	3-Hydroxy-3-methylglutaricacid	5.18	C_6_H_10_O_5_	[M − H]^−^	161.04561	161.04591	1.86	59.01392, 99.04560	Database	+	+
22	Organic acids	Methylsuccinic acid	5.44	C_5_H_8_O_4_	[M − H]^−^	131.03498	131.03531	2.52	87.04517	Database	+	+
23	Other	Porphobilinogen	5.59	C_10_H_14_N_2_O_4_	[M − H]^−^	225.08808	225.08815	0.31	71.01408, 59.01390	Database	+	+
24	Organic acids	Pantothenic acid	5.78	C_9_H_17_NO_5_	[M + H]^+^	220.11795	220.11795	0.00	202.10733, 184.09677	Database	+	+
25	Organic acids	3,4-Dihydroxyphenylacetic acid	5.81	C_8_H_8_O_4_	[M − H]^−^	167.03498	167.03497	−0.06	123.04532	Database	+	+
26	Organic acids	3-Hydroxy-3-methylbutanoic acid	6.46	C_5_H_10_O_3_	[M − H]^−^	117.05572	117.05588	1.37	115.04015	Database	+	+
27	Other	DL-Mandelic acid	6.81	C_8_H_8_O_3_	[M − H]^−^	151.04007	151.04005	−0.12	107.05027	Database	+	−
28	Organic acids	5-Hydroxyindole-3-acetic acid	6.89	C_10_H_9_NO_3_	[M + H]^+^	192.06552	192.06572	1.04	146.06006, 147.06815	Database	+	−
29	Other	Levetiracetam *	7.44	C_8_H_14_N_2_O_2_	[M + H]^+^	171.11280	171.11287	0.41	126.09128, 89.07086	Database	+	+
30	Other	Salicylic acid	7.73	C_7_H_6_O_3_	[M − H]^−^	137.02442	137.02446	0.29	93.03464	Database	+	+
31	Other	2-Isopropylmalic acid	7.91	C_7_H_12_O_5_	[M − H]^−^	175.06120	175.06129	0.51	115.04014, 85.06591	Database	+	+
32	Organic acids	Terephthalic acid	8.56	C_8_H_6_O_4_	[M − H]^−^	165.01930	165.01939	0.55	121.02962	Database	+	+
33	Other	L-Iditol	8.79	C_6_H_14_O_6_	[M − H]^−^	181.07176	181.07173	−0.17	71.01383, 101.02444, 89.02440	Database	+	+
34	Organic acids	benzoic acid *	9.18	C_7_H_6_O_2_	[M − H]^−^	121.02950	121.02961	0.91	93.03453	Database	+	+
35	flavonoid	Daidzin *	9.86	C_21_H_20_O_9_	[M − H]^−^	415.10346	415.10333	−0.30	253.05060	Database	+	+
36	flavonoid	Glycitin	10.09	C_22_H_22_O_10_	[M + H]^+^	447.12857	447.12881	0.53	285.07568	Database	+	−
37	Amide	Phenacetin *	10.33	C_10_H_13_NO_2_	[M + H]^+^	180.10191	180.10208	0.94	162.09142	Database	+	−
38	flavonoid	Puerarin	10.67	C_21_H_20_O_9_	[M − H]^−^	415.10345	415.10315	−0.74	267.07166	Database	+	−
39	Organic acids	Mesaconicacid	10.78	C_5_H_6_O_4_	[M − H]^−^	129.01933	129.01918	−1.18	57.03464	Database	+	+
40	flavonoid	Genistin *	11.23	C_21_H_20_O_10_	[M − H]^−^	431.09837	431.09869	0.74	268.03806	Database	+	+
41	Other	Azelaic acid	12.03	C_9_H_16_O_4_	[M − H]^−^	187.09758	187.09756	−0.12	125.09720, 97.06583	Database	+	+
42	flavonoid	Daidzein *	12.48	C_15_H_10_O_4_	[M + H]^+^	255.06519	255.06508	−0.43	227.07021, 199.07533, 137.02328, 91.05417	Database, literature [18]	+	+
43	flavonoid	Chrysin	12.54	C_15_H_10_O_4_	[M − H]^−^	253.05063	253.05075	0.47	209.06131	Database	+	+
44	flavonoid	Glycitein *	12.68	C_16_H_12_O_5_	[M − H]^−^	283.06119	283.06122	0.08	268.03796	Database	+	+
45	flavonoid	4’,7-Dihydroxyflavanone	12.70	C_15_H_12_O_4_	[M + H]^+^	257.08084	257.08072	−0.47	91.05438, 81.03360	Database	+	+
46	flavonoid	Genistein *	13.43	C_15_H_10_O_5_	[M + H]^+^	271.06009	271.06030	0.74	253.04997, 243.06535, 215.07024, 197.05974, 169.06479, 153.01862	Database, literature [19]	+	+
47	flavonoid	Naringenin *	13.48	C_15_H_12_O_5_	[M − H]^−^	271.06120	271.06152	1.18	151.00377, 119.05029, 107.01385	Database	+	+
48	flavonoid	Fisetin	13.49	C_15_H_10_O_6_	[M − H]^−^	285.04046	285.04071	0.88	135.00868	Database	+	+
49	flavonoid	Kaempferide *	13.52	C_16_H_12_O_6_	[M − H]^−^	299.05611	299.05618	0.23	285.09622	Database	+	+
50	Other	Cycluron	14.12	C_11_H_22_N_2_O	[M + H]^+^	199.18049	199.18042	−0.35	72.04436	Database	+	+
51	Terpenoids	Armillarinin	14.23	C_24_H_29_O_7_Cl	[M + H]^+^	465.16746	465.16757	0.24	199.01570	Database, literature [20]	+	−
52	Terpenoids	Soyasaponin I	14.45	C_48_H_78_O_18_	[M + H]^+^	943.52609	943.526	−0.10	441.37283, 599.39441, 797.46838	Database	+	+
53	Sphingolipid	2-Amino-1,3,4-octadecanetriol	14.70	C_18_H_39_NO_3_	[M + H]^+^	318.30027	318.30026	−0.03	300.29114	Database	+	+
54	Terpenoids	Armillarilin	14.89	C_24_H_30_O_7_	[M + H]^+^	431.20643	431.20645	0.05	165.05458	Database, literature [20]	+	−
55	Steroid	Estriol	15.4	C_18_H_24_O_3_	[M + H]^+^	289.17982	289.17978	−0.14	159.08081	Database	+	−
56	Terpenoids	Armillarin	15.83	C_24_H_30_O_6_	[M + H]^+^	415.21152	415.21149	−0.07	165.05475	Database, literature [21]	+	+
57	Terpenoids	Dehydroeburicoic acid	16.23	C_31_H_48_O_3_	[M + H]^+^	469.36762	469.36755	−0.15	451.35825	Database	+	−
58	Terpenoids	Armillaribin	16.72	C_24_H_28_O_5_	[M + H]^+^	397.20095	397.20105	0.25	215.14302, 165.05461	Database, literature [22]	+	+
59	Other	Piptamine	16.81	C_23_H_41_N	[M + H]^+^	332.33118	332.33112	−0.18	240.26860, 91.05413	Database	+	+
60	flavonoid	ar-Turmerone	16.87	C_15_H_20_O	[M + H]^+^	217.15869	217.15857	−0.55	91.05415	Database	+	−
61	Terpenoids	Armillaricin	16.92	C_24_H_27_O_5_Cl	[M + H]^+^	431.16198	431.16202	0.09	215.14302, 199.01567, 187.14812	Database, literature [22]	+	−
62	Other	Cetrimonium	16.97	C_19_H_41_N	[M + H]^+^	284.33118	284.33118	0.01	60.08073	Database	+	−
63	Other	Coriolic acid	17.22	C_18_H_32_O_3_	[M − H]^−^	295.22787	295.22809	0.75	277.21747, 195.13911	Database	+	+
64	Terpenoids	Melleolide	17.24	C_23_H_28_O_6_	[M + H]^+^	401.19587	401.1958	−0.17	233.15363	Database, literature [22]	+	−
65	Other	Phthalic anhydride	18.29	C_8_H_4_O_3_	[M + H]^+^	149.02332	149.02328	−0.27	121.02829	Database	+	−
66	Esters	Dioctyl phthalate	19.81	C_24_H_38_O_4_	[M + H]^+^	391.28427	391.28436	0.19	71.08540	Database	+	+
67	Amide	Linoleamide	19.96	C_18_H_33_NO	[M + H]^+^	280.26349	280.26355	0.21	263.23700, 245.22643	Database, literature [23]	+	+
68	Sphingolipid	D-Sphingosine	20.09	C_18_H_37_NO_2_	[M + H]^+^	300.28971	300.28976	0.17	282.27960, 283.26321	Database	+	+
69	Esters	1-Linoleoyl glycerol	20.51	C_21_H_38_O_4_	[M + H]^+^	355.28429	355.284	−0.81	91.05740	Database	+	+
70	Amide	Palmitamide	21.14	C_16_H_33_NO	[M + H]^+^	256.26349	256.26324	−0.98	74.06001, 69.06985, 57.06992, 55.05424	Database	+	+
71	Organic acids	Linoleic acid	21.47	C_18_H_32_O_2_	[M − H]^−^	279.23295	279.23306	0.38	261.22192	Database, literature [24]	+	+
72	Amide	Oleamide	21.49	C_18_H_35_NO	[M + H]^+^	282.27914	282.279	−0.50	265.25250	Database, literature [23,25]	+	+
73	Organic acids	Palmitoleic Acid	21.65	C_16_H_30_O_2_	[M + H]^+^	255.23186	255.23196	0.40	237.22130	Database	+	+
74	Steroid	Ergosterol *	21.85	C_28_H_44_O	[M + H]^+^	397.34649	397.34674	0.63	379.33572	Database	+	−
75	Steroid	Ergosterol endoperoxide	21.97	C_28_H_44_O_3_	[M + H]^+^	429.33632	429.33643	0.25	411.32599, 393.31552	Database, literature [26]	+	−
76	Organic acids	Palmitic acid *	22.58	C_16_H_32_O_2_	[M − H]^−^	255.23295	255.23296	0.03	256.23624	Database, literature [25]	+	+
77	Organic acids	Oleic acid	22.89	C_18_H_34_O_2_	[M − H]^−^	281.24860	281.24869	0.31	282.25192	Database	+	+
78	Esters	Linolenic acid ethyl ester	23.57	C_20_H_34_O_2_	[M + H]^+^	307.26316	307.26355	1.28	123.11696	Database	+	−
79	Esters	Ethyl oleate	26.10	C_20_H_38_O_2_	[M + H]^+^	311.29446	311.29456	0.33	265.25259	Database	+	+

Note: Compounds with “*” indicate that they have been verified by a standard; those without “*” are speculative. “FJH” Fermented asparagus Tuder Halimasch powder. “FJQ” GTHP before fermentation. “+” indicates inclusion; “−” indicates exclusion.

**Table 2:** Results of similarity evaluation of 10 batches of GTHP after fermentation.

ID	Similarity	ID	Similarity
S1	0.971	S6	0.984
S2	0.992	S7	0.975
S3	0.986	S8	0.999
S4	0.978	S9	0.999
S5	0.998	S10	0.996

**Table 3:** Results of the linear survey of uracil, guanosine, adenosine, 5-HMF, daidzin, genistin, glycitein, daidzein, genistein and ergosterol.

Compound	Regression Equation	R^2^	Linear Range (µg/mL)
Uracil	Y = 9,189,836.9096 X − 8756.4263	0.9998	0.99–32.84
Guanosine	Y = 6,907,785.2972 X − 6209.2408	0.9998	1.03–34.34
Adenosine	Y = 6,569,226.0327 X − 10,826.0629	0.9996	1.77–58.82
5-HMF	Y = 5,677,358.0948 X − 12,014.4694	0.9996	2.22–74.12
Daidzin	Y = 9,104,442.8249 X − 2582.8260	0.9997	0.30–10.15
Genistin	Y = 18,562,283.4133 X − 5566.5236	0.9997	0.27–8.92
Glycitein	Y = 13,180,712.1644 X − 5008.3407	0.9997	0.40–13.24
Daidzein	Y = 18,397,395.8876 X − 17,212.7445	0.9998	1.08–36.03
Genistein	Y = 29,448,803.7592 X − 3572.4452	0.9997	0.76–25.29
Ergosterol	Y = 2,134,505.6341 X − 2630.5954	0.9999	1.91–63.73

**Table 4:** Results of recovery determination for 10 compounds.

Compound	Sampling Volume/g	Sample Content/mg	Addition/mg	Measured Amount/mg	Recovery Rate/%	Average Recovery Rate/%	RSD/%
Uracil	0.5001	0.1754	0.1620	0.3335	97.59	99.34	1.48
	0.5004	0.1755	0.1620	0.3382	100.43		
	0.5003	0.1755	0.1620	0.3389	100.86		
	0.5001	0.1754	0.1620	0.3381	100.43		
	0.5002	0.1755	0.1620	0.3336	97.59		
	0.5001	0.1754	0.1620	0.3360	99.14		
Guanosine	0.5001	0.1093	0.0997	0.2066	97.59	98.06	2.12
	0.5004	0.1093	0.0997	0.2071	98.09		
	0.5003	0.1093	0.0997	0.2085	99.50		
	0.5001	0.1093	0.0997	0.2088	99.80		
	0.5002	0.1093	0.0997	0.2032	94.18		
	0.5001	0.1093	0.0997	0.2082	99.20		
Adenosine	0.5001	0.2323	0.2085	0.4367	98.03	98.77	1.81
	0.5004	0.2325	0.2085	0.4421	100.53		
	0.5003	0.2324	0.2085	0.4386	98.90		
	0.5001	0.2323	0.2085	0.4321	95.83		
	0.5002	0.2324	0.2085	0.4423	100.67		
	0.5001	0.2323	0.2085	0.4380	98.66		
5-HMF	0.5001	0.2062	0.2230	0.4252	98.21	98.26	2.87
	0.5004	0.2063	0.2230	0.4365	103.23		
	0.5003	0.2063	0.2230	0.4219	96.68		
	0.5001	0.2062	0.2230	0.4227	97.09		
	0.5002	0.2062	0.2230	0.4276	99.28		
	0.5001	0.2062	0.2230	0.4182	95.07		
Daidzin	0.5001	0.0033	0.0055	0.0086	96.36	98.18	2.34
	0.5004	0.0033	0.0055	0.0088	100.00		
	0.5003	0.0033	0.0055	0.0087	98.18		
	0.5001	0.0033	0.0055	0.0089	101.82		
	0.5002	0.0033	0.0055	0.0086	96.36		
	0.5001	0.0033	0.0055	0.0086	96.36		
Genistin	0.5001	0.0022	0.0035	0.0056	97.14	96.67	1.21
	0.5004	0.0022	0.0035	0.0055	94.29		
	0.5003	0.0022	0.0035	0.0056	97.14		
	0.5001	0.0022	0.0035	0.0056	97.14		
	0.5002	0.0022	0.0035	0.0056	97.14		
	0.5001	0.0022	0.0035	0.0056	97.14		
Glycitein	0.5001	0.0152	0.0311	0.0462	99.68	98.34	2.09
	0.5004	0.0152	0.0311	0.0465	100.64		
	0.5003	0.0152	0.0311	0.0453	96.78		
	0.5001	0.0152	0.0311	0.0449	95.50		
	0.5002	0.0152	0.0311	0.0455	97.43		
	0.5001	0.0152	0.0311	0.0463	100.00		
Daidzein	0.5001	0.0617	0.0757	0.1364	98.68	99.10	1.79
	0.5004	0.0617	0.0757	0.1368	99.21		
	0.5003	0.0617	0.0757	0.1342	95.77		
	0.5001	0.0617	0.0757	0.1377	100.40		
	0.5002	0.0617	0.0757	0.1376	100.26		
	0.5001	0.0617	0.0757	0.1376	100.26		
Genistein	0.5001	0.0683	0.0841	0.1497	96.79	99.07	1.66
	0.5004	0.0683	0.0841	0.1532	100.95		
	0.5003	0.0683	0.0841	0.1524	100.00		
	0.5001	0.0683	0.0841	0.1528	100.48		
	0.5002	0.0683	0.0841	0.1507	97.98		
	0.5001	0.0683	0.0841	0.1509	98.22		
Ergosterol	0.5001	0.2453	0.2313	0.4677	96.15	99.65	2.60
	0.5004	0.2455	0.2313	0.4748	99.14		
	0.5003	0.2454	0.2313	0.4857	103.89		
	0.5001	0.2453	0.2313	0.4759	99.70		
	0.5002	0.2454	0.2313	0.4727	98.27		
	0.5001	0.2453	0.2313	0.4784	100.78		

**Table 5:** Determination of 10 components in GTHP samples before and after fermentation (mg/g).

Sample	Uracil	Guanosine	Adenosine	5-HMF	Daidzin	Genistin	Glycitein	Daidzein	Genistein	Ergosterol
S1	0.3304	0.2040	0.4271	0.4582	0.0085	0.0061	0.0434	0.1560	0.1697	0.4910
S2	0.3018	0.1987	0.4512	0.4015	0.0087	0.0061	0.0462	0.1731	0.1838	0.4917
S3	0.2935	0.2095	0.4741	0.4140	0.0092	0.0063	0.0466	0.1773	0.1859	0.4841
S4	0.3369	0.1445	0.3113	0.4427	0.0069	0.0053	0.0336	0.1125	0.1262	0.4504
S5	0.3417	0.1806	0.3569	0.4508	0.0075	0.0057	0.0369	0.1285	0.1397	0.4523
S6	0.3151	0.1948	0.4415	0.4418	0.0086	0.0063	0.0447	0.1631	0.1728	0.4956
S7	0.3508	0.2185	0.4646	0.4123	0.0066	0.0043	0.0304	0.1234	0.1365	0.4906
S8	0.2187	0.2547	0.6121	0.3363	0.0112	0.0088	0.0613	0.2439	0.2288	0.4980
S9	0.2400	0.2158	0.5083	0.3465	0.0096	0.0071	0.0526	0.2023	0.1971	0.4666
S10	0.1814	0.2617	0.6307	0.2788	0.0117	0.0080	0.0625	0.2570	0.2375	0.4878
S11	0.0327	0.0244	0.0308	/	0.0524	0.0394	0.0156	0.0237	0.0190	/

Note: S1–S10 denote post-fermentation GTHP, and S11 denotes pre-fermentation GTHP.

**Table 6:** Experimental controls, lot numbers, and manufacturers.

Control	Batch Number	Manufacturing Company
Cytidine	B20073	Shanghai Yuanye Biotechnology Co., Shanghai, China
Betaine	PS012048	Chengdu Pusi Biotechnology Co., Chengdu, China
Nicotinic acid	PS020097	Chengdu Pusi Biotechnology Co., Chengdu, China
2,6-Dihydroxypurine	PS020191	Chengdu Pusi Biotechnology Co., Chengdu, China
Uridine	887-200202	China National Institute for the Control of Pharmaceutical and Biological Products, Beijing, China
Adenosine	110879-200202	China National Institute for the Control of Pharmaceutical and Biological Products, Beijing, China
Guanosine	PS010291	Chengdu Pusi Biotechnology Co., Chengdu, China
Uracil	U13135C30	Chengdu Pusi Biotechnology Co., Chengdu, China
Ascorbic acid	J04A10R84808	Shanghai Yuanye Biotechnology Co., Shanghai, China
5-Methyl-2-furaldehyde	J23S6X3622	Shanghai Yuanye Biotechnology Co., Shanghai, China
5-hydroxymethylfurfural	H81835D5F	Shanghai Jiji Biochemical Technology Co., Shanghai, China
D-phenylalanine	H06A8H33287	Shanghai Yuanye Biotechnology Co., Shanghai, China
Levetiracetam	PS230518-15	Chengdu Pusi Biotechnology Co., Chengdu, China
Benzoic acid	PS161011-06	Chengdu Pusi Biotechnology Co., Chengdu, China
Daidzin	PS011899	Chengdu Pusi Biotechnology Co., Chengdu, China
Phenacetin	PS230518-16	Chengdu Pusi Biotechnology Co., Chengdu, China
Genistin	111709-200501	China National Institute for the Control of Pharmaceutical and Biological Products, Beijing, China
Daidzein	B20227	Shanghai Yuanye Biotechnology Co., Shanghai, China
Glycitein	PS011931	Chengdu Pusi Biotechnology Co., Chengdu, China
Genistein	111704-200501	China National Institute for the Control of Pharmaceutical and Biological Products, Beijing, China
Naringenin	PS010355	Chengdu Pusi Biotechnology Co., Chengdu, China
Kaempferide	PS011599	Chengdu Pusi Biotechnology Co., Chengdu, China
Ergosterol	111845-202105	China National Institute for the Control of Pharmaceutical and Biological Products, Beijing, China
Palmitic acid	PS001166	Chengdu Pusi Biotechnology Co., Chengdu, China

## Data Availability

All the data in the manuscript are available upon reasonable request.
